# Experimental Study of the Thermally Grown Oxide and Interface of Thermal Barrier Coatings Using TEM In-Situ Heating

**DOI:** 10.3390/nano12224020

**Published:** 2022-11-16

**Authors:** Hongye Zhang, Runlai Peng, Jiaye Zhao, Chao Fan, Wei Feng, Zhanwei Liu

**Affiliations:** 1 School of Technology, Beijing Forestry University, Beijing 100083, China; 2 School of Aerospace Engineering, Beijing Institute of Technology, Beijing 100081, China; 3 Institute of Flexible Electronics Technology of THU, Jiaxing 314006, China; 4 Beijing Institute of Spacecraft Environment Engineering, Beijing 100094, China

**Keywords:** TBCs, real time, TGO, strain/stress measurement

## Abstract

Thermal barrier coating (TBC) materials play important roles in gas turbine engines to protect the Ni-based superalloys from high-temperature airflow damage. In this work, the nano-mechanism of TBC failure is analyzed. A scanning transmission electron microscopy-energy dispersive spectrometer (STEM-EDS)-based analysis method was used to study the influence of element migration on the deformation behavior of the bond–coat (BC) layer during heating. The content of elements in the same region varied greatly at different temperatures, which could prove the contribution of element migration to the deformation of the BC layer. TEM in-situ heating experiments were designed and carried out to study the deformation behavior near the ceramic topcoat (TC)/thermally grown oxide (TGO) and the TGO/BC interface. The TC/TGO interface was deformed violently during heating, and obvious deformation occurred at 100 °C, while the TGO/BC interface was relatively stable. A subset geometric phase analysis method was used for full field-strain measurement. The strain value near the TGO/BC interface was relatively small and did not change significantly at lower temperatures. The TC/TGO interface is more unstable and easier to deform than the TGO/BC interface. The stress and strain evolution in the internal region of TGO at high temperatures was quantitatively analyzed. The TGO layer has a tensile stress of GPa magnitude along the interface direction at the peak position, and the shear stress is small.

## 1. Introduction

Thermal barrier coatings (TBCs) are one of the most advanced high-temperature protective coatings available, featuring low thermal conductivity, good high-temperature chemical stability, scouring resistance and thermal insulation [1,2,3,4,5,6]. TBCs are generally composed of three layers of material: superalloy substrate [7,8,9], bond coat (BC) and ceramic topcoat (TC). TBCs typically serve in high-temperature cyclic environments, which may result in sintering effects, phase changes, and crack generation and extension. These phenomena are induced by growth stress, internal deformation, temperature-dependent material transport mechanism, and other factors. Therefore, it is necessary to characterize and understand the microstructural change mechanism of TBCs at high temperatures, from a nano view.

After a period of working in a high-temperature environment, areas between the TC and BC will form an oxide layer (thermally grown oxide, TGO) several µm thick, consisting mainly of α-Al_2_O_3_, which has a low diffusion rate of oxygen ions and strong adhesion. Studies [10] have shown that the formation and growth of TGO on the bonding layer surface is a typical metal surface oxidation process, which is in accordance with the well-known Wagnerian oxidation parabolic law. The failure of TBCs often occurs at the TC/TGO and TGO/BC interfaces, which is a key factor affecting the thermodynamic properties and durability of TBCs [11,12,13]. There is large residual stress [14] at the TC/TGO and TGO/BC interfaces, and the occurrence and development of damage in the form of microcracks in TBCs have multiple different forms [3]. The reason is that TGO generates large residual stresses during the processes of generation and growth, resulting in stress concentrations that lead to cracking, spalling, and eventually failure of the coating structure. Under the coupled service environment of heat and force, the mismatch of thermodynamic parameters of each material layer can cause thermal mismatch stresses in the TBCs matrix system [15,16]. The thermodynamic parameters of ceramic layers are generally measured at room temperature [17,18,19,20]. Miller’s [17] research shows that the stress–strain relationship of the coating is nonlinear when it is calcined at a high temperature. Studies on the formation of residual stresses in TGO and the development of stress concentrations have mainly focused on macroscopic and microscopic aspects, while there are relatively few studies on the finer nanoscale. Studies [21,22] observed the scale structure of TGO but did not analyze and study the stress and strain distribution and its evolution within the TGO.

Due to the high temperatures caused by the gas, the ceramic layer material undergoes sintering and phase changes. High-resolution electron microscopy, such as X-ray diffraction techniques (XRD) and SEM, is the main tool for microstructural changes within ceramic layer characterization [23,24]. During the service process of TBCs, the ceramics undergo phase transformation as the temperature is continuously changed. Yttria partially stabilized zirconia (YSZ) may transition from the initial tetragonal phase to the monoclinic phase, and the accompanying bulk and martensite effects produce large phase transformation stresses, which accelerate the failure of TBCs [25]. Some studies [26,27] focus on the magnitude and distribution of TGO stress in EB-PVD TBCs after high-temperature thermal cycling, and the relationship with the duration of thermal cycling. In another area of application, thermal coatings, which are thermally sprayed intermetallics that thermally stimulate structure evolution, have also been researched [28]. In recent years, researchers have gradually recognized that microscopic interfaces are always the source of crack development and coating failure through finite element simulations of the evolution of the microstructure of TBC structures [29,30,31].

Although many studies have been conducted on the mechanical properties, damage, and life prediction of TBCs, and preliminary exploratory studies have been conducted to explore the generation and growth of TGO, the mechanism underlying the failure behavior of TGO/TBC and TGO/BC interfaces remains unclear. Based on the scale of TGO, it is necessary to study such materials at the even-smaller nanoscale, which is the key location that failure occurs. The purpose of the present study is to investigate the nano-mechanisms of TBC failure in the TBC structure of an aero engine blade. Specifically, the online analysis method based on scanning transmission electron microscopy-energy dispersive spectrometer (STEM-EDS, Bruker super-X, Bruker, Billerica, GER) was used to study the influence of element migration on the deformation behavior of the BC layer during heating. TEM in-situ heating experiments were designed and carried out to study the deformation behavior near the TC/TGO interface. The high-temperature deformation behavior near the TGO/BC interface was quantitatively analyzed, and the in-situ online stress and strain evolution in the internal region of TGO at high temperature was quantitatively analyzed.

## 2. Materials and Methods

### 2.1. Material and Specimen

**Figure 1:**
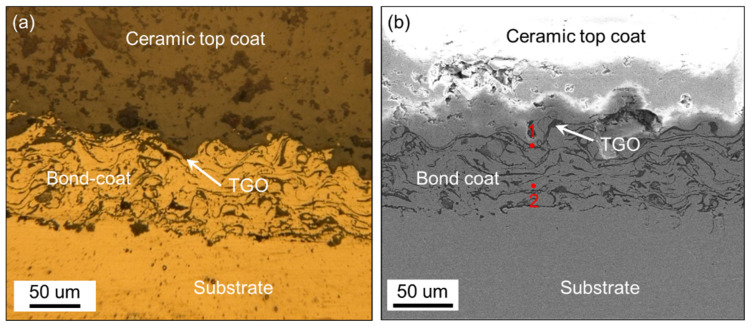
Sectional image of the TBCs sample captured by (**a**) super-depth microscope and (**b**) SEM.

The base material used in this investigation was a commercial Ni-based super alloy (GH4169) with geometrical dimensions of 25 × 10 × 2 mm^3^. Firstly, the surface of samples was ground with sandpaper to remove oxides, ultrasonically cleaned with alcohol for degreasing, and blasted to increase the adhesive strength of coatings. The TBCs were then air-plasma sprayed onto the Ni-based substrate (APS-2000, Aviation Industry Corporation of China CO., Ltd., Beijing, China) at the Institute of Process Engineering, Chinese Academy of Science, in Beijing. The TC consisted of 45.5% Ni, 23% Co, 25% Cr, 6% Al, and 0.5% Y. The BC was a commercial 8 wt.% YSZ powder (Beijing Hang Bai Chuan Technology Center, Beijing, China), which features a hollow spheroidized structure with a diameter of 15–70 μm. The air-plasma spraying parameters were as follows: the current is 600 A, the voltage is 70 V, the spraying distance is 100–110 mm, and the powder feed rate is 30 g/min. The thicknesses of the fabricated BC and TC from bottom to top were about 100 and 500 μm, respectively. The produced TBC samples were cyclic heated in a resistance furnace (KSL-1200X-J, HEFEI KEJING MATERIALS TECHNOLOGY CO., Ltd., Hefei, China). The heating process was in three steps: 0.5 h heating up to 1150 °C, 10 h thermal insulation at 1150 °C, and natural cooling to room temperature. After six instances of cyclic oxidation, TGO was observed using an SEM (FEI Quanta FEG 450, FEI, OR, USA) and a super-depth microscope (VHX-500FE, KEYENCE, Osaka, Japan), with a TGO thickness of about 1~3 um (see Figure 1). Table 1 shows the element content at the two different points in Figure 1, measured by EDS. The main elements at point 1 are Al and O. Al_2_O_3_ is the main component of TGO. Point 2 does not contain O, indicating the main component of the BC layer.

**Figure 2:**
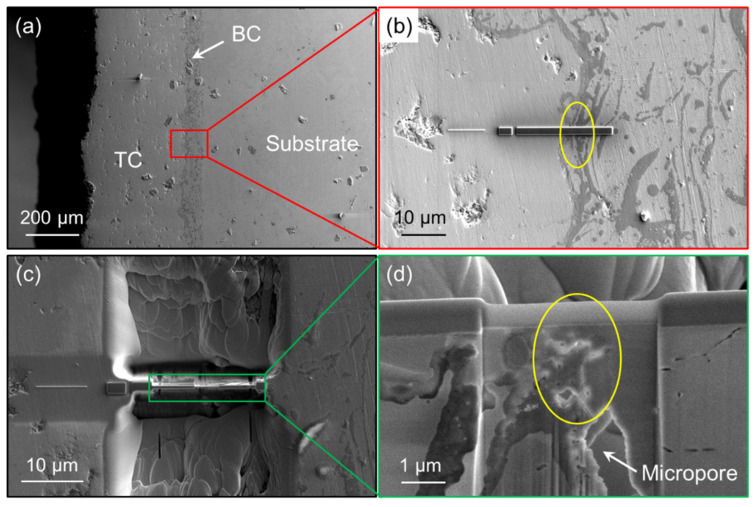
TBCs TEM sample preparation process using FIB-EB double-beam system: (**a**) Sample overview; (**b**) Depositing Pt; (**c**) Front view of the prepared TEM sample; and (**d**) Side view of the TEM sample’s thin area.

Figure 2 shows the process of TBC high-temperature TEM samples prepared by the FIB-EB double-beam system. Firstly, the sample surface was treated, and then the sample was mounted on the sample stage. In order to reduce the damage of FIB to the sample, a thin layer of Pt was deposited by electron beam in the region of interest (ROI), and a rectangle of 8 × 2 μm^2^ was generally deposited (the length of a TEM sample is always 5~6 μm, as shown in the subgraph in the upper right corner of Figure 2). By selecting different FIB beams, the ROI was gradually separated from the bulk sample, and the TEM sample was reduced to a thickness of several hundred nanometers as a whole. According to need, a length of 3~4 μm was selected in the middle of the sample to further reduce the thickness, by adjusting the FIB beam and incident angle, as shown in the subgraph of the second row in Figure 2. Finally, the ROI of the sample was reduced to a thickness of about tens of nanometers. In order to prevent Ga^+^ ions from being implanted into the sample, it is necessary to observe the state of Pt deposited on the surface of the sample by SEM. After successful thinning of the ROI, the bottom and sides of the slice sample were cut off with FIB, and the extracted sample was fixed on a heating chip to form a TEM sample for observation. The specimen was examined using a FEI Tecnai Remote (Tecnai G2 F20, FEI, OR, USA) transmission electron microscope operating at 200 kV, employing a combination of bright-field (BF) and high-resolution transmission electron microscopy (HRTEM) images. EDS microanalysis was also performed in this TEM.

### 2.2. TEM In-Situ Heating

**Figure 3:**
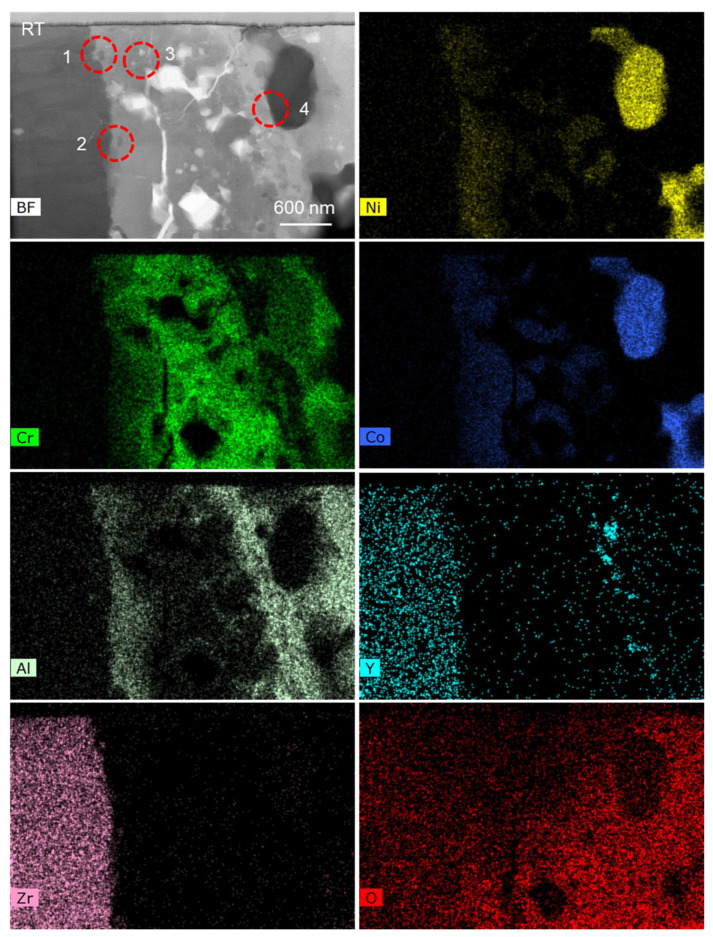
STEM-EDS elemental maps of the TBCs sample with magnification of 20,000.

The TEM sample was transferred to the heating chip after preparation, which is an MEMS-based single-tilt TEM sample heating and biasing holder (FEI NanoEx-i/v, FEI, OR USA). Then, the TEM sample was fixed on the heating chip by depositing Pt on the edge of the sample, where no Pt was deposited on the upper region (observation area) near the sample. So, the upper region was free to expand, without any external constraints. During the heating and cooling stage, the speed of temperature variation was 1 °C/s. At the heating stage, in-situ HRTEM observation was made at room temperature (RT), 150 °C, 300 °C, 400 °C, 500 °C, 600 °C, 700 °C, 800 °C, and back to RT. The literature [3,14] shows that the TC/TGO and TGO/BC interfaces are the weak points inside the TBCs. Here, TEM in-situ heating was used to study the influence of thermal load on different positions inside the TBCs. Figure 3 shows the STEM-EDS maps of the observed thin area. The TC layer can be clearly distinguished, while the boundary between the BC layer and the TGO is not obvious. The BC layer is in the more rightward area, and the enrichment of Al elements very near the TC layer is not obvious. According to the element distribution, region 1 contains the TC/TGO interface, region 2 is mainly inside the TGO, region 3 contains Ni, Co, and Cr elements, and its surroundings are mainly alumina. So, region 3 contains the TGO/BC interface.

**Figure 4:**
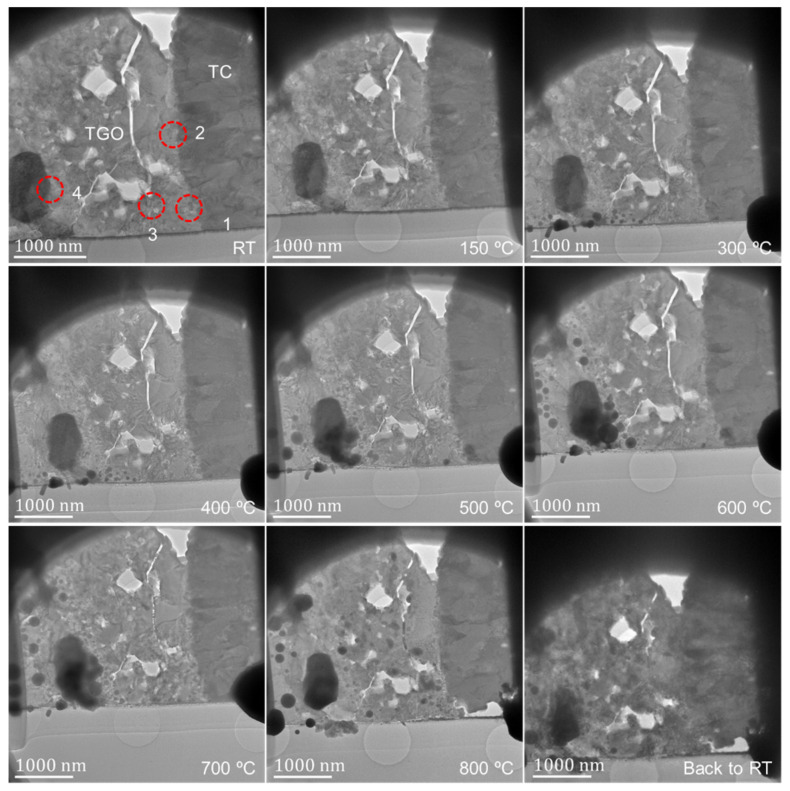
Morphology of the heated sample at different temperatures.

Figure 4 shows the TEM bright-field images of the observation area at different temperatures, and the corresponding four regions in Figure 3 are marked. Due to the different positions of the TEM mode and the STEM mode signal collector, the morphology images in Figure 3 and Figure 4 have an inverted corresponding relationship. With the increase in temperature, the different grains in the TBCs have different changes. At 300 °C, there is obvious gallium ion precipitation, and the phenomenon of grain fusion and growth in the TC layer is more obvious. From the morphology, the phenomenon of sample melting occurs in the observation area after exceeding 500 °C. When the temperature exceeds 500 °C, clear lattice fringes cannot be obtained in different regions under high-resolution observation. When the temperature is up to 800 °C then drops to RT, the sample recrystallizes. Compared with the sample state before heating, the crystal structure of the observation area has undergone dramatic changes.

### 2.3. Subset Geometric Phase Analysis for Strain Measurement

The geometric phase analysis (GPA) method, introduced by Hÿtch [32,33] is successfully applied in the displacement/strain field analysis of crystal structures at the nanoscale. The so-called S-GPA, inspired by GPA, was improved by Liu [34], where the windowed Fourier transform was added in the transforming process. There have also been some developments and important applications of the GPA method in recent years [35,36,37,38,39]. The core theoretical formula in S-GPA [34] is as follows.

The two-dimensional windowed Fourier transform (2D-WFT) is:

(1) $$Q(\mu ,\upsilon ,\xi ,\eta )=\int_{-\infty }^{\infty }\int_{-\infty }^{\infty }q(x,y)g(x-\mu ,y-\upsilon )\exp\{-i\xi x-i\eta y\}dxdy$$

where Q(μ,υ,ξ,η) represents the windowed Fourier spectrum;ξ and η represent the frequency components in the *x* and *y* directions, respectively; g is a window function representing the reciprocal lattice vector of the lattice; (μ,υ) is the coordinate of the center of the window and the target window changes with different pairs of μ and υ.

The 2D inverse WFT is:

(2) $$\hat{q}(x,y)=\frac{1}{4\pi^{2}}\int_{-\infty }^{\infty }\int_{-\infty }^{\infty }\int_{-\infty }^{\infty }\int_{-\infty }^{\infty }\bar{Q}(\mu ,\upsilon ,\xi ,\eta )g(x-\mu ,y-\upsilon )\times \exp\{i\xi x+i\eta y\}d\xi d\eta d\mu d\upsilon$$

with

(3) $$\bar{Q}(\mu ,\upsilon ,\xi ,\eta )=\left\{ \begin{array}{l} Q(\mu ,\upsilon ,\xi ,\eta )\;\;,\;\;\;\;\;\;\;\;\;\;\;\;\left|Q(\mu ,\upsilon ,\xi ,\eta )\right|/max(\left|Q(\mu ,\upsilon ,\xi ,\eta )\right|)\;\;\geq Thr\\ 0\;\;\;\;\;\;\;\;\;\;\;\;\;\;\;\;\;\;\;\;,\;\;\;\;\;\;\;\;\;\;\;\;\left|Q(\mu ,\upsilon ,\xi ,\eta )\right|/max(\left|Q(\mu ,\upsilon ,\xi ,\eta )\right|)\;\;<Thr \end{array}\right.$$

where $\bar{Q}(\mu ,\upsilon ,\xi ,\eta )$ represents the filtered frequency spectrum;  |Q(μ,υ,ξ,η)| represents the power of Q(μ,υ,ξ,η); the mostly used Gaussian window function g is divided by $\sqrt{\pi \sigma_{x}\sigma_{y}}$ for normalization; Thr is the preset threshold.

The wrapped phase can be obtained through the simple arctangent function $\hat{q}(x,y)$. Using the phase unwrapping technique, the original phase field and the phase difference can be obtained. The displacement/strain can be calculated through the following matrix form equation:

(4) $$\left(\begin{array}{cc} \varepsilon_{xx} & \varepsilon_{xy}\\ \varepsilon_{yx} & \varepsilon_{yy} \end{array}\right)=\left(\begin{array}{cc} \frac{\partial u(x)}{\partial x} & \frac{\partial u(x)}{\partial y}\\ \frac{\partial u(y)}{\partial x} & \frac{\partial u(y)}{\partial y} \end{array}\right)=-\frac{1}{2\pi }{\left(\begin{array}{cc} g_{1x} & g_{1y}\\ g_{2x} & g_{2y} \end{array}\right)}^{-1}\left(\begin{array}{cc} \frac{\partial P_{g_{1}}}{\partial x} & \frac{\partial P_{g_{1}}}{\partial y}\\ \frac{\partial P_{g_{2}}}{\partial x} & \frac{\partial P_{g_{2}}}{\partial y} \end{array}\right)$$

where the subscripts *x* and *y* represent the *x* and *y* directions. u(x) and u(y) are the displacements in the *x* and *y* directions, $P_{g}$ is the phase in the image, $\varepsilon_{xx}$ and $\varepsilon_{yy}$ are the direct strains, $\varepsilon_{xy}$ and $\varepsilon_{yx}$ are the shear strains, respectively. During strain measurement, the possible influence caused by the sample preparation process is all but ignored.

## 3. Results and Discussion

### 3.1. Experimental Study on Element Migration Based on STEM-EDS Analysis

**Figure 5:**
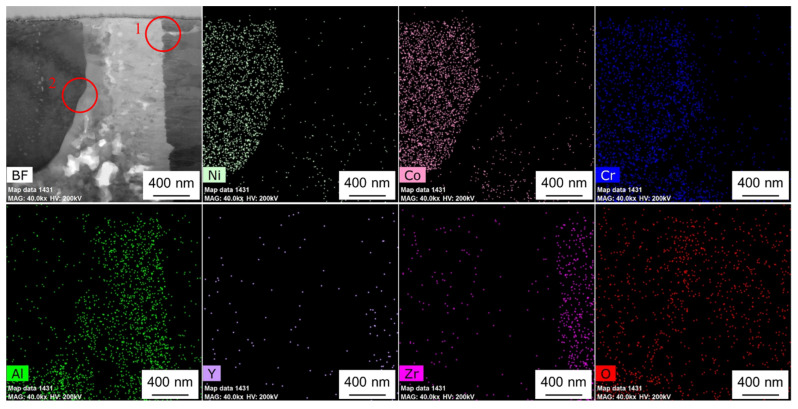
STEM-EDS elemental maps of the observation area.

**Figure 6:**
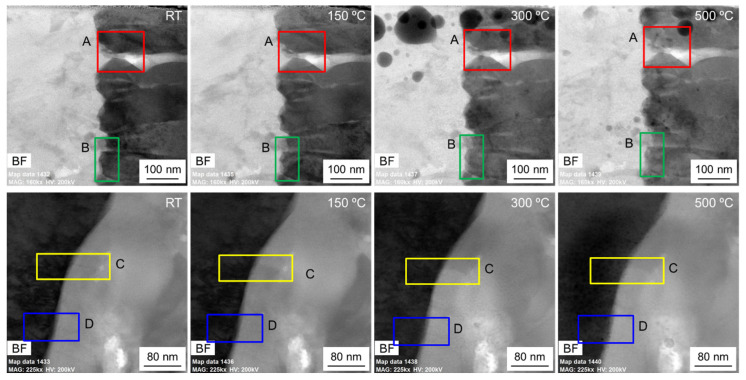
STEM-BF images of the observation area at different temperatures.

In our previous work [5], based on the SEM-EDS analysis, the migration of elements in the BC layer was studied, and the deformation of the BC layer in the thermal shock experiment was studied to some extent. In the STEM mode, EDS was used to detect the element distribution in real time under heating conditions. Figure 5 shows the STEM-EDS map of the observation area. According to the element distribution, TC, TGO, and BC layers can be easily determined. The elemental contents of regions 1 and 2 at RT, 150 °C, 300 °C, and 500 °C were measured. Region 1 contains the TC/TGO interface and is close to the free surface of the sample, and region 2 contains the TGO/BC interface. Figure 6 is the BF image of two regions in Figure 5 at different temperatures in the STEM mode. The four regions marked by A, B, C, and D in Figure 5 are the positions for statistical analysis of element content. At the same time, it can be found that at 300 °C, obvious gallium ion precipitation occurs near the free surface of the sample.

During the experiment, the EDS analysis had the same scanning time at different temperatures. Table 2 and Table 3 show the proportion of different elements near the TC/TGO interface and TGO/BC interface at different temperatures, respectively. According to the two tables, the content of Al element increases with increasing temperature near the TC/TGO interface only in region B, whereas the law is not obvious in region A near the TC/TGO interface. At the same time, the element distribution of regions C and D in the TGO/BC interface region also has no obvious law, but there are certain content changes. When in-situ heating in TEM, the sample is in an anaerobic environment. It has been demonstrated that the activity of the element increases with temperature change, and the content of different elements at the same position changes, a phenomenon known as element migration. The experiment here is carried out at the nanoscale, and the element change can only represent the local micro-region. The increase of Al content in region B is in good agreement with the existing literature [40,41], while the change of Al content in region A is not obvious. The selected sub-region A is too close to the TC layer, and the Al content is too small. The element migration activity in TBCs during heating is active. Although it is different from the aerobic heating environment in the literature [5], it can also explain the role of element migration in the evolution of the strain state of the BC layer during thermal shock to some extent.

In this experiment, the sample was placed in the TEM in advance for more than 12 h to minimize the influence of sample vibration on the measurement results. In any case, thermal radiation will affect the EDS signal collection. TEM samples are photographed during heating, and there is a high probability of sample drift. The larger the magnification, the more obvious the drift. Sample drift and the limitation of the EDS working environment may introduce some measurement errors. These factors may be the reason why the measurement results are not regular here.

### 3.2. TC/TGO Interface Analysis

**Figure 7:**
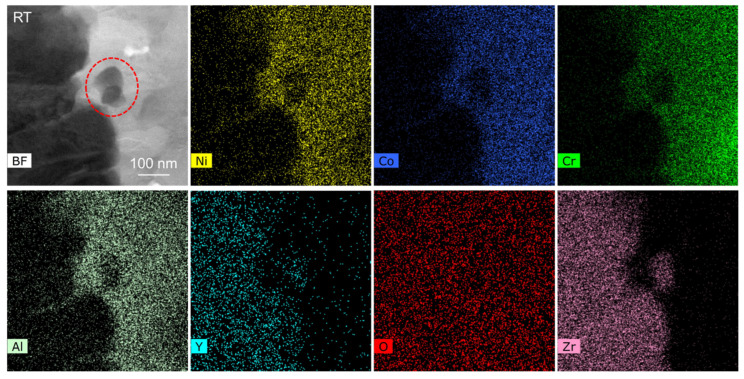
STEM-EDS maps of region 1 in Figure 3 and Figure 4.

Figure 7 shows the STEM-EDS map of region 1 in Figure 3 and Figure 4. According to different element distributions, it can be determined that there is a TC/TGO interface in this region. The area selected by the red dotted line contains a zirconia particle. Region 2 in Figure 3 and Figure 4 also contains a zirconia particle, which is used as a marker to observe the behavior of the TC/TGO interface region at different temperatures.

**Figure 8:**
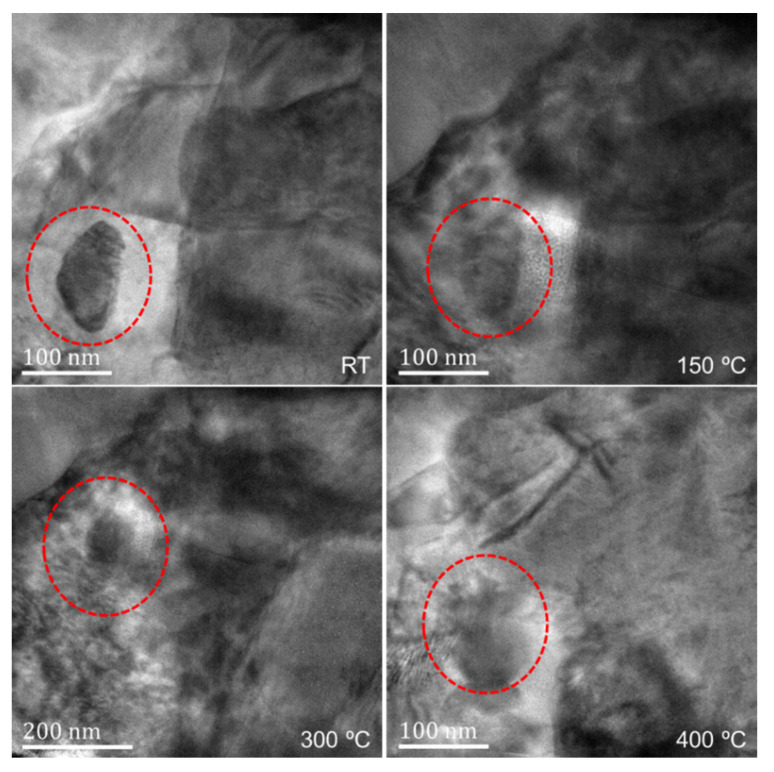
Morphology of TC/TGO interface at different temperatures.

Figure 8 shows the morphology evolution of region 2 in Figure 3 and Figure 4 at different temperatures. The position of the zirconia particle is marked, and there is a TC/TGO interface at the right edge of the particles to the right of about 70 nm. Unfortunately, clear lattice fringe images cannot be obtained by adjusting the crystal band axis at high resolution, so specific quantitative calculations cannot be performed. Through the directional analysis of the change of the TC/TGO interface morphology image, it can be found that with the increase of temperature, the TC/TGO interface morphology has changed obviously (150 °C). The change of image gray scale shows that different lattice structures have a large relative deformation. With the further increase in temperature, the zirconia particle changes greatly, and the shape becomes blurred. After more than 400 °C, the accurate position of the zirconia particle cannot be accurately found. The morphology of the TC/TGO interface shows that during the heating process, the deformation near this type of interface is large and complex.

### 3.3. TGO/BC Interface Analysis

**Figure 9:**
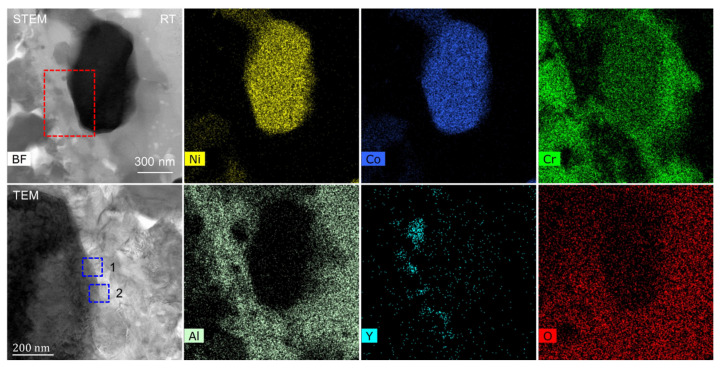
STEM-EDS maps of region 4 in Figure 3 and Figure 4.

Figure 9 shows the STEM-EDS map of region 4 in Figure 3 and Figure 4, and the TEM image of the red dashed rectangle in the STEM image is also shown. The TGO/BC interface in this region can be determined based on the different element distributions and the grayscale distribution of the TEM image.

**Figure 10:**
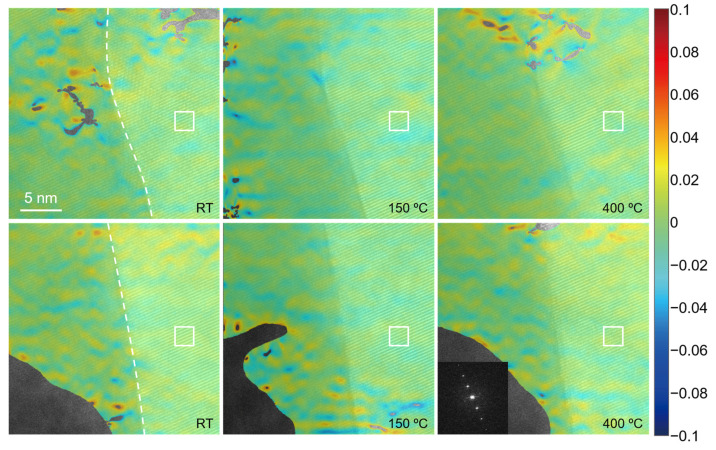
Strain distribution near the TGO/BC interface at different temperatures.

Figure 10 shows the high-resolution images of the blue dotted rectangular regions 1 and 2 in Figure 9 at different temperatures and the strain field distribution in the main direction of the fringe calculated by the S-GPA method. The first row of the image corresponds to region 1, and the second row corresponds to region 2. Due to the limitation of the single-tilt sample holder, only the unidirectional clear lattice fringe was obtained. The diffraction spectrum at 400 °C in region 2 is given, and the diffraction spectrum of other high-resolution images is consistent with its form. According to the gray level of the image and the distribution of the EDS maps, the interface at the two regions is determined to be the position shown by the white dotted line in the figure. Due to the lack of diffraction information, the specific phase represented by the lattice on both sides of the interface cannot be calculated. In the S-GPA calculation, the white rectangular region in the image is selected to construct the reference phase, and strain results in the corresponding region, where the lattice fringes cannot be distinguished in the high-resolution image, are removed. From the strain field distribution of the two regions, it can be found that there is no obvious non-uniform deformation or strain concentration at the TGO/BC interface in the observation area. The TGO/BC interface in the observation area is relatively stable when heated to 400 °C. However, when the temperature is above 500 °C, no clear lattice fringes can be obtained in the two observed regions. Under the action of high temperature, the TGO/BC interface also changes greatly. Two possible reasons are considered here. One is that the crystal structure of the observation area has changed greatly during the heating process. The second is the material melting and even amorphization in the observation area due to the high temperature.

It can be found that after the TBC structure is in service, the thermal deformation of the TC/TGO interface during the subsequent heating process is greater. Compared with the TGO/BC interface structure, the TC/TGO interface structure is more unstable and easier to deform and damage under high temperatures. The TGO/BC interface also has a large structural change when the temperature is high. The extreme value of the strain is relatively small. The TC/TGO interface is less stable than the TGO/BC interface. It is more likely to deform and form stress concentrations in high temperature service. It is more likely to cause damage failure at the TC/TGO interface, which would lead to the spalling failure of the coating structure. The cracks generated at the two interfaces are the two main forms of cracks in the TBCs. This further supplements the conclusion in the literature [3] that the TC/TGO and TGO/BC interfaces are the weak positions in the TBC structure.

### 3.4. TGO Internal Analysis

**Figure 11:**
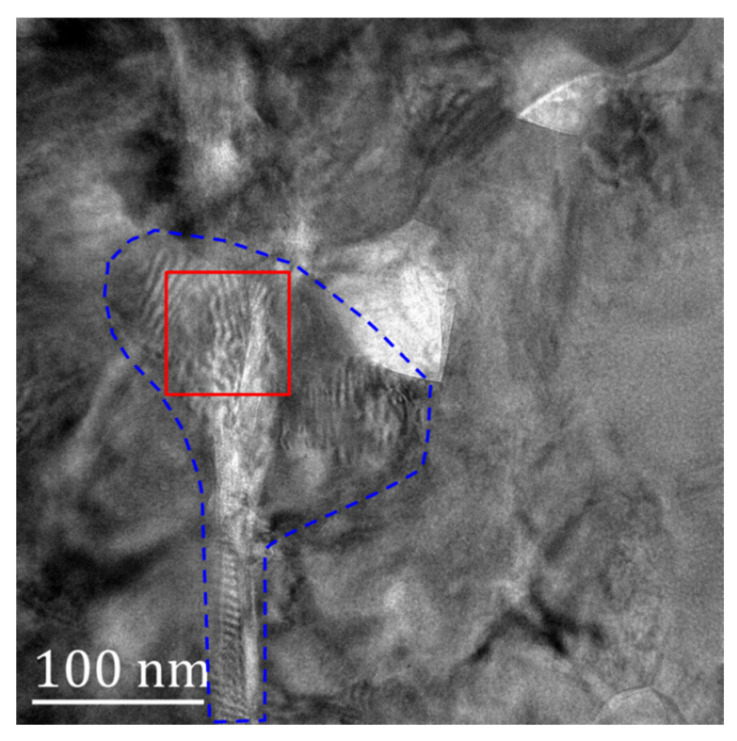
TEM image of TGO.

Studies have shown that there is a large stress in the TGO, and the presence of TGO greatly affects the durability of the TBC structure. The peeling failure of the coating often occurs near the interface of the TGO [42,43]. Here, the stress distribution inside the TGO would be explored at the nanoscale. The enlarged image of region 3 in Figure 3 and Figure 4 is shown in Figure 11. Irregular Moiré fringes appear in the blue dotted region. The lattice fringes and Moiré fringes in the red rectangular area are further observed.

**Figure 12:**
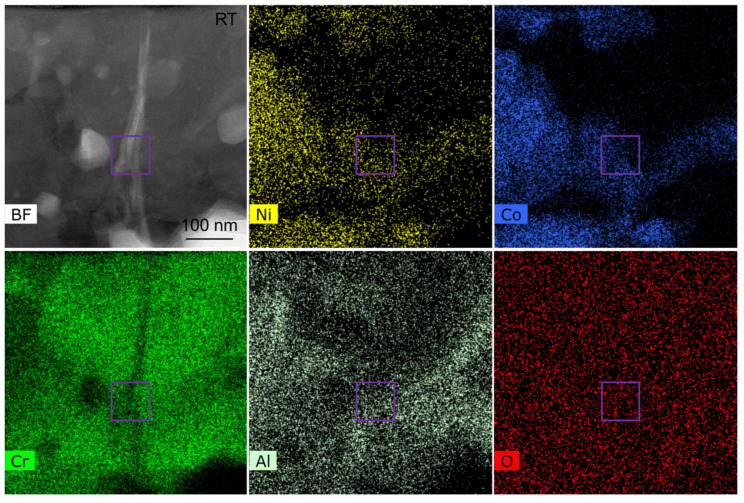
STEM-EDS maps of the internal region of TGO.

The STEM-EDS map of the corresponding region in Figure 11 is shown in Figure 12. The element distribution in the red rectangular area in Figure 11 corresponds to the purple region in Figure 12. There are five elements of Ni, Co, Cr, Al, and O. Therefore, it is determined that the region is indeed inside the TGO. From the large-scale element distribution, it can also be found that the element distribution in each region inside the TGO is uneven, which would have different effects on the stress distribution in different regions inside the TGO.

**Figure 13:**
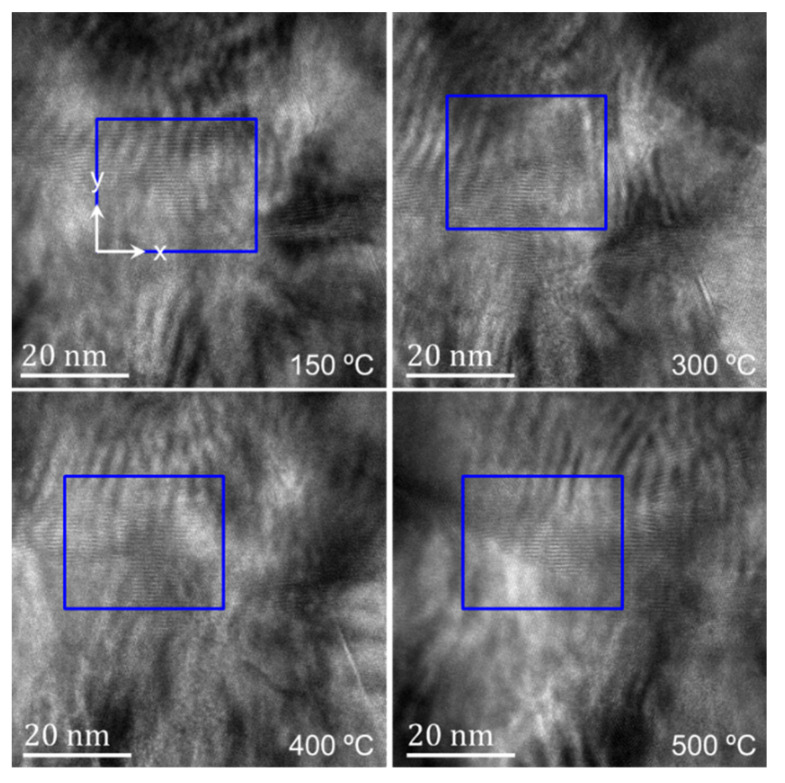
HRTEM images of TGO at different temperatures.

The high-resolution images of the red rectangular region in Figure 11 at 150 °C, 300 °C, 400 °C, and 500 °C were obtained during the in-situ heating. As shown in Figure 13, it can be found that the Moiré fringes vary greatly at different temperatures and the clarity of different positions varies differently, indicating that there is also a more severe deformation inside the TGO during heating. At the same time, the high-resolution image contains lattice fringes in different directions, meaning that clear atomic lattice structures can be found in different regions. However, due to the complex sample conditions and high-temperature experimental conditions, the lattice structure in some regions is not obvious, and the atomic lattice fringes in individual directions can only be accurately judged. It is necessary to be cautious when calculating the strain field of lattice fringes in different directions for stress calculation.

**Figure 14:**
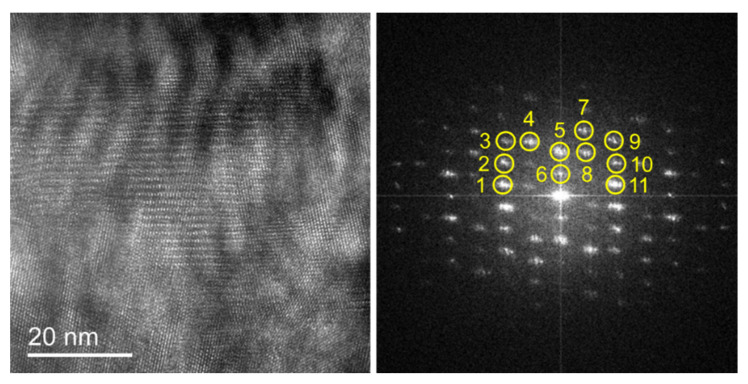
HRTEM image and its corresponding diffraction spectrum in the observation region of TGO at 150 °C.

In order to further accurately calculate the strain and stress inside the TGO, the crystal structure at 150 °C was first analyzed, as shown in Figure 14. From the diffraction spectrum, it can be found that there may be a variety of structures in the observation area. Through calculation, the plane spacing corresponding to the diffraction points 1 to 11 in the diffraction spectrum is 0.2966 nm, 0.2452 nm, 0.2090 nm, 0.2669 nm, 0.3845 nm, 0.7554 nm, 0.2363 nm, 0.3354 nm, 0.2104 nm, 0.2567 nm, and 0.2957 nm. Through the calibration method of the polycrystalline electron diffraction pattern, the high-resolution image cannot accurately determine the phase type of the region. Ni, Co, Cr, Al, and O can form a variety of different compounds. Therefore, TGO is regarded as a unified and uniform whole and its specific phase type is not determined in the subsequent calculation.

**Figure 15:**
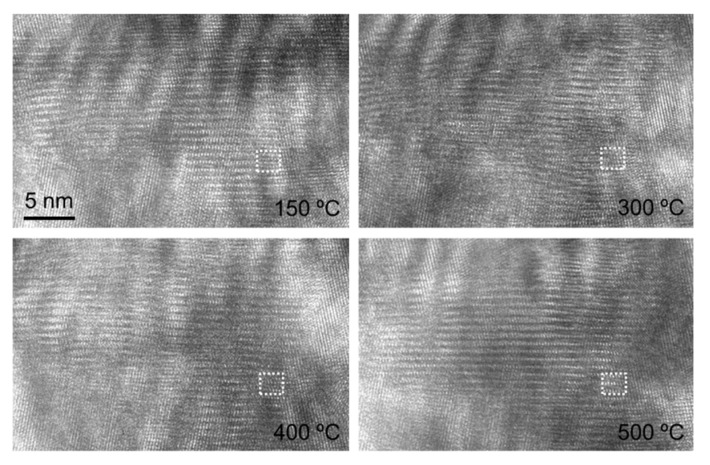
HRTEM images of the same region in TGO at different temperatures.

A high-resolution image of the same region within the TGO at different temperatures shown in Figure 13 is found by means of feature recognition, as shown in Figure 15, where the region in the white dotted box is used to construct a reference phase in the strain calculation of S-GPA. By analyzing the high-resolution images at different temperatures, it is found that the lattice fringes corresponding to diffraction points 1 and 11 are clear and distinguishable in a large range, which is conducive to quantitative calculation, while many pseudo-dislocation results will appear when the lattice fringes in other directions are used for calculation.

**Figure 16:**
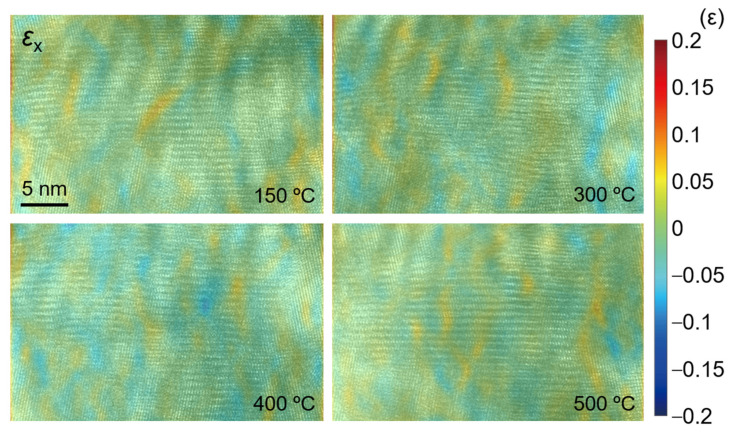
Calculated strain field *ε_xx_*.

**Figure 17:**
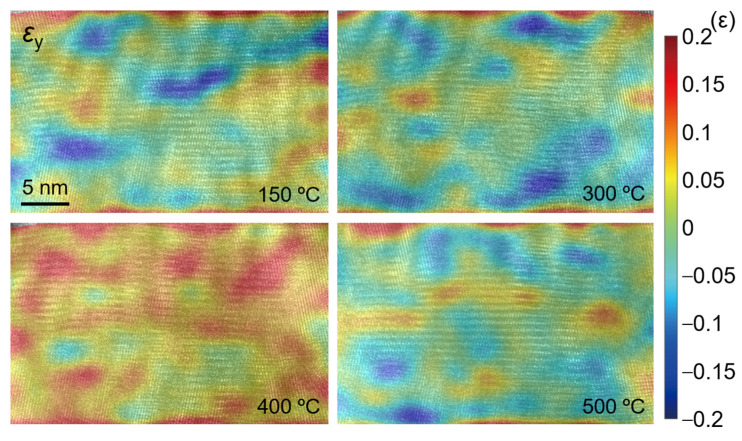
Calculated strain field *ε_yy_*.

**Figure 18:**
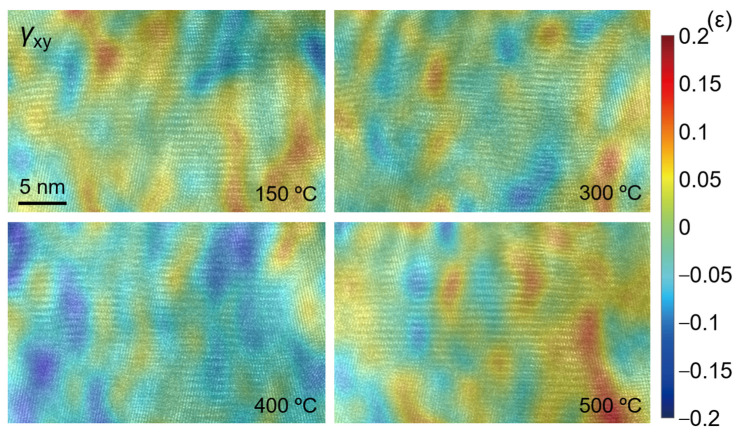
Calculated strain field *ε_xy_*.

Figure 16, Figure 17 and Figure 18 show the strain fields in the *x* and *y* directions and shear strain fields at different temperatures in the observation area calculated by the S-GPA method. From the distribution of strain fields, there is no obvious law of strain-field evolution in the same region at different temperatures. The strain value in the *x* direction is relatively small and uniform, the strain value and shear strain value in the *y* direction are relatively large, and the *y* direction is mainly tensile strain. There is a strain concentration phenomenon at different positions, indicating that the energy contained there is high and unstable, which is a relatively weak area in TGO. The strain distribution shows that the TGO in the observation area is mainly affected by the tensile strain and shear strain in the *y* direction.

In this research, the TEM sample can be regarded as a thin plate with a nanometer thickness, which conforms to the assumption of the plane stress problem. The stress distribution at different temperatures in the observation area can be analyzed by using the obtained strain field.

(5) $$\left\{ \begin{array}{l} \varepsilon_{x}=\frac{1}{E}\left(\sigma_{x}-\nu \sigma_{y}\right)\\ \varepsilon_{y}=\frac{1}{E}\left(\sigma_{y}-\nu \sigma_{x}\right)\\ \gamma_{xy}=\frac{2\left(1+\nu \right)}{E}\tau_{xy} \end{array}\right.$$

here, $\varepsilon_{x}$, $\varepsilon_{y}$, $\sigma_{x}$, and $\sigma_{y}$ are strain and stress in the *x* and *y* directions respectively. $\gamma_{xy}$ is shear strain. $\tau_{xy}$ is shear stress. E is elastic modulus of the material. ν is Poisson ratio. Table 4 shows the elastic constants of TGO obtained from the literature.

Previously, it has been pointed out that the specific phase in the observation area cannot be judged, so its elastic constant cannot be used for stress calculation. Here, the elastic constant of TGO in the literature is directly used for calculation, and it is regarded as an isotropic organic whole without considering the differences in different regions at the micro-nano level. It can be found from Table 4 that the elastic modulus and Poisson’s ratio of TGO are different in different studies, which is related to many factors, such as the preparation process and heating conditions of the material. Here, the elastic constant of TGO is selected as: *E* = 350 GPa, *ν* = 0.25.

**Figure 19:**
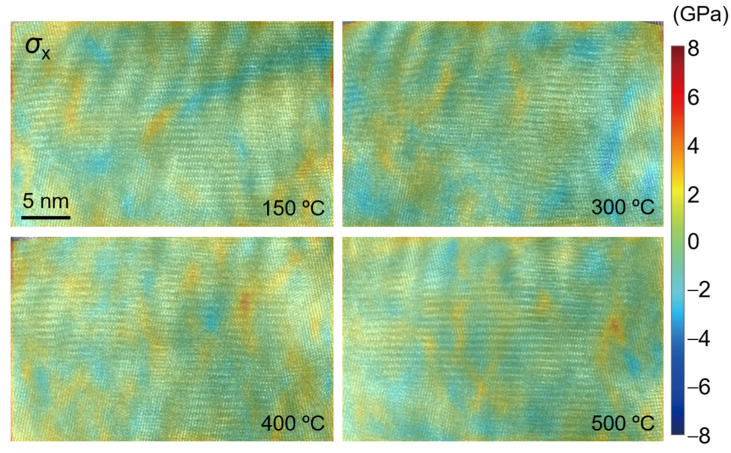
Calculated stress field *σ_x_*.

**Figure 20:**
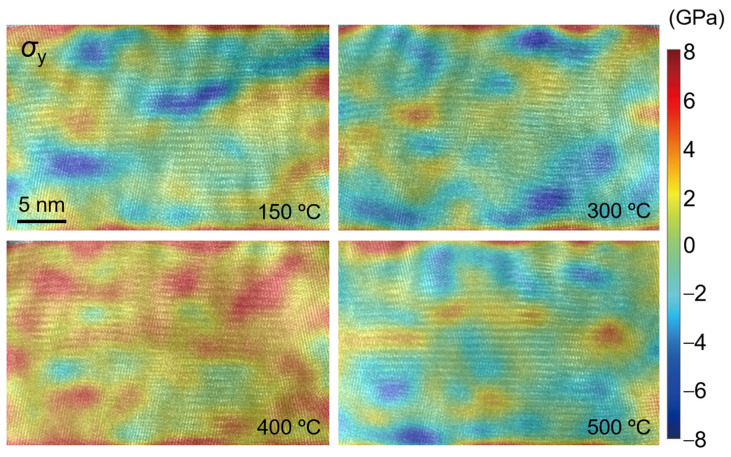
Calculated stress field *σ_y_*.

**Figure 21:**
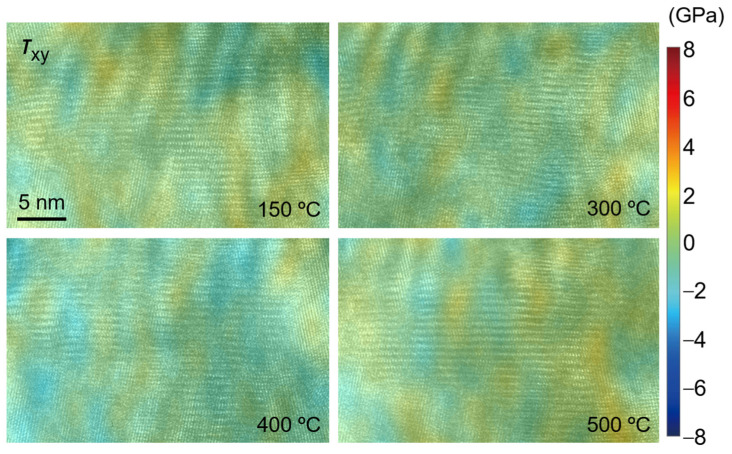
Calculated shear stress field.

The stress distribution at different temperatures in the observation area of TGO is calculated by using Equation (5), as shown in Figure 19, Figure 20 and Figure 21. Similar to the distribution of the strain field, the stress value in the *y* direction is large, and it is mainly tensile stress at 400 °C. The values of stress and shear stress in the *x* direction are relatively small, which are about one order of magnitude smaller than the stress value in the *y* direction. The stress in the *x* direction is the stress in the out-of-plane direction. The absolute value of stress is basically less than 1.5 GPa. Its irregular evolution will affect the deformation and failure of the TC/TGO and TGO/BC interfaces. In the *y*-direction stress field, the stress amplitude is about 6 GPa. A uniform color-bar can distinguish the distribution and difference of stress in different directions. Researchers [51] pointed out that when the temperature is less than 900 °C, the yield stress of TGO is 10 GPa. Therefore, when there is no external load in the heating experiment in this study, TGO can exist stably despite some changes at different temperatures.

As shown in Figure 2, Figure 3 and Figure 4 and Figure 13, the position of the TGO in this study is just at the peak position of the TGO structure on a larger three-dimensional scale. The observation position is in the yellow elliptical region of the subgraph in the second column of Figure 2 (the upper region of the microhole in the subgraph in the lower right corner of Figure 2). In this study, the *x* direction is set to be perpendicular to the extension direction of the TC/TGO interface, while the *y* direction is parallel to the spreading direction of the TC layer (parallel to the extension direction of the TC/TGO interface), which is subjected to a tensile stress of the order of GPa in the *y* direction. This is also consistent with the conclusion of the existing theoretical analysis [10,11], which also proves the correctness of its theoretical calculation from the level of nano-experimental research. At the same time, some scholars [52] measured the average stress in TGO under different heat treatment times by photoluminescence piezoelectric spectroscopy. The stress was in the order of GPa and the maximum was greater than 4 GPa. Other studies [14,51,53] have also shown that the stress value in TGO is on the order of GPa. Using near-field optical microscopy [53], simulation [51], etc., some studies [54] have shown that the stress value in TGO is distributed around hundreds of megapascals. The problem of the internal stress distribution of TGO is that we need to consider a variety of different working conditions, such as heating time, whether there is online measurement, different element content, and other factors for comprehensive calculation and evaluation, to finally determine the influence of TGO on TBC structure during service and eliminate and optimize the structure and material of TBCs.

## 4. Conclusions

TBC materials were subjected to relevant experimental research and analysis. The main findings in the current work can be summarized as follows:

1. The migration of different elements was in-situ studied in the BC layer. The content of elements in the same region varied greatly, which could prove the contribution of element migration to the deformation of the BC layer.
2. The evolution of the TC/TGO interface and the TGO/BC interface during heating was obtained. The TC/TGO interface was deformed violently during heating, and obvious deformation occurred at 100 °C, while the TGO/BC interface was relatively stable. From the strain analysis near the high-resolution image interface, the strain value near the TGO/BC interface was relatively small and did not change significantly at lower temperatures. The TC/TGO interface is unstable and easier to deform than the TGO/BC interface. It is a weak area in the TBC structure, and its deformation and failure are more likely to cause the coating to fall off.
3. The in-situ heating experiment reveals the evolutionary behavior of strain and stress in TGO. The TGO layer does have a tensile stress of GPa magnitude along the interface direction at the peak position, and the shear stress is small.

## Figures and Tables

**Table 1:** Content of different elements at the two points in Figure 1 (wt.%).

	Ni	Co	Cr	Al	Y	O
Point 1	2.01	1.88	5.45	45.84	7.91	36.93
Point 2	41.41	36.46	20.53	1.51	0.09	—

**Table 2:** Proportion of different elements near TC/TGO interface at different temperatures (wt.%).

	Region A				Region B			
	RT	150 °C	300 °C	500 °C	RT	150 °C	300 °C	500 °C
O	7.18	4.81	7.35	6.68	8.59	4.40	6.75	5.04
Al	9.13	8.88	6.04	9.50	6.07	8.79	10.83	11.64
Zr	29.48	86.31	86.61	83.82	85.34	86.80	82.42	83.32

**Table 3:** Proportion of different elements near TGO/BC interface at different temperatures (wt.%).

	Region C				Region D			
	RT	150 °C	300 °C	500 °C	RT	150 °C	300 °C	500 °C
O	7.84	8.24	7.19	4.70	9.25	3.62	3.81	6.02
Al	4.64	3.31	4.36	2.89	1.94	3.81	3.03	1.59
Cr	33.45	34.81	28.69	30.53	28.82	28.69	28.60	31.31
Co	29.02	32.98	29.59	33.19	33.26	36.17	32.35	37.88
Ni	26.03	20.67	30.17	28.68	26.74	27.71	32.22	23.21

**Table 4:** The elastic constants of TGO in the literature.

Literature	[44]	[45]	[23]	[46]	[47]	[48]	[49]	[50]
*E* (GPa)	400	350	400	200–390	400	350	400	380
*ν*	/	0.25	0.23	/	0.25	0.2	0.2	0.2

## Data Availability

Not applicable.
